# Supplementary material for: Epigenetic Regulation of Neuregulin-1 Tunes White Adipose Stem Cell Differentiation
Source: Cells. 2020 May 7;9(5):1148. doi: 10.3390/cells9051148 (PMC7290571; doi:10.3390/cells9051148)
Supplement: Supplementary file 1 [file cells-09-01148-s001.pdf]

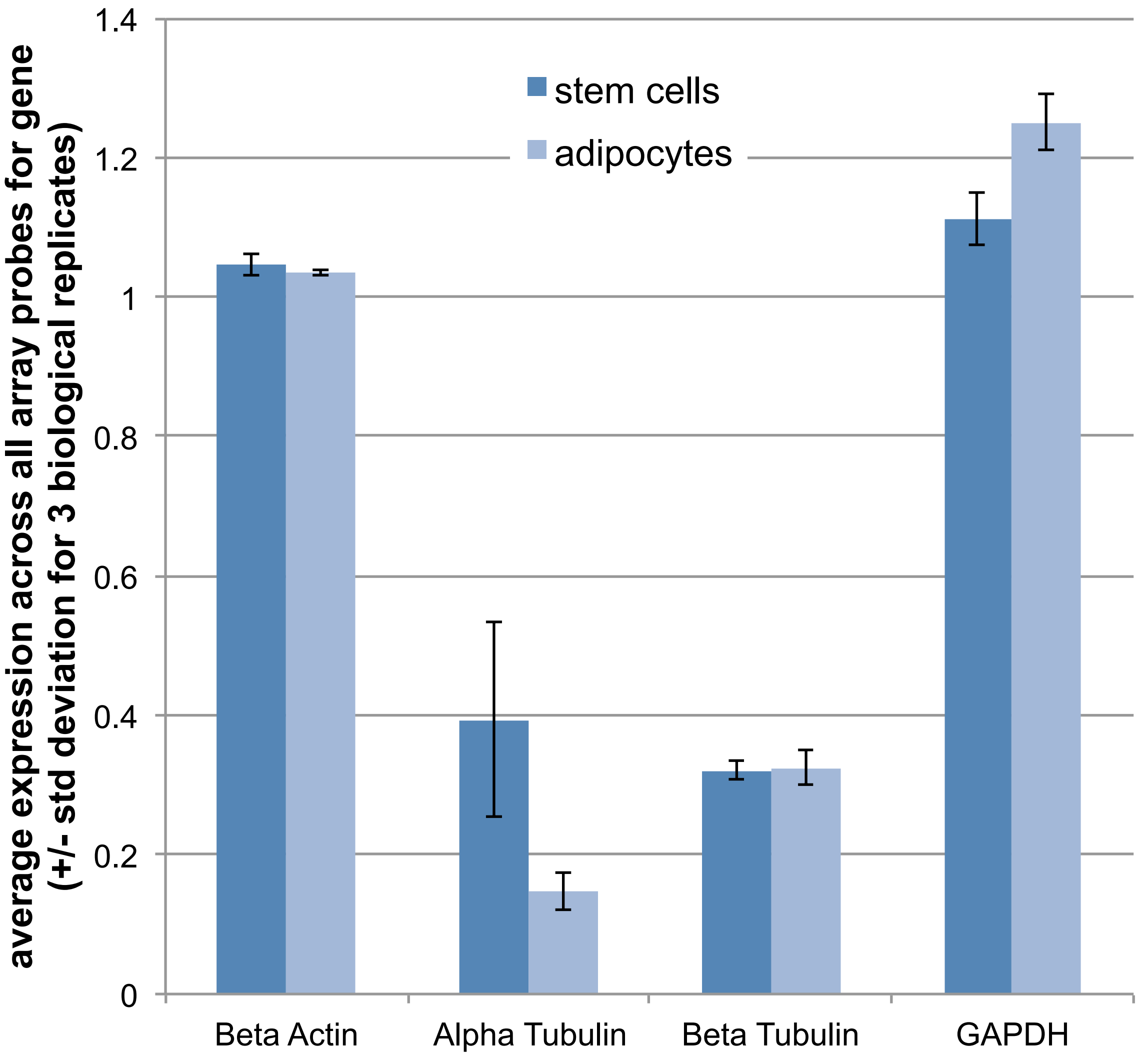

Figure S1. Analysis of housekeeping gene expression and adipose stem cell differentiation. Average expression was calculated as genometric mean of three biological replicates.

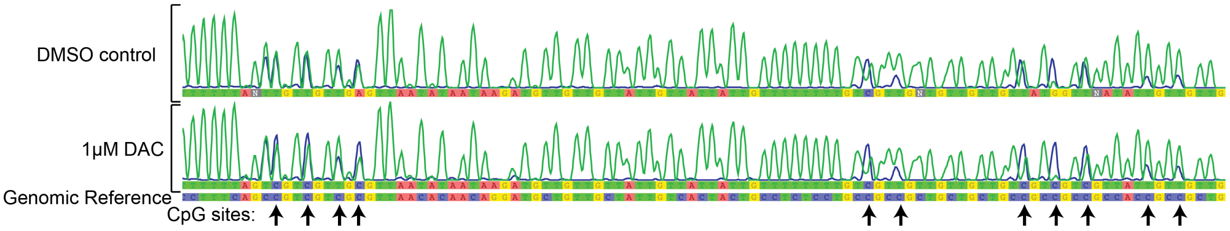

Figure S2. Bisulfite PCR analysis of NRG1 Type III CpG island. Bisulfite treatment causes cytosine (C, blue) to sequence as thymidine (T, green). Methylation is detected as retention of cytosine (blue traces) relative to T (green traces) on chromatograms (for clarity, G and A traces omitted). Note that 1 μm DAC does not demethylate this locus. Arrows mark CpG sites where methylation can occur.

Supplemental Table 1. All genes significantly upregulated or downregulated by DAC in volcano plot.

Transcript Cluster ID	Fold Change	ANOVA p-value (	Gene Symbol	Description
11752634_x_at	18.58	0.000084	KRT8	keratin 8, type II
11758298_x_at	18.25	0.000018	KRT8	keratin 8, type II
11756989_x_at	15.22	0.000071	KRT8	keratin 8, type II
11758184_x_at	13.85	0.00002	KRT8	keratin 8, type II
11717386_s_at	13.22	0.000681	MT1G	metallothionein 1G
11758188_x_at	11.85	0.00006	KRT8	keratin 8, type II
11758301_x_at	11.51	0.000118	KRT8	keratin 8, type II
11758183_x_at	11.23	0.000159	KRT8	keratin 8, type II
11727248_a_at	8.85	0.000055	MYH3	myosin, heavy chain 3, skeletal muscle, embryonic
11733121_s_at	8.33	0.000016	DAZL	deleted in azoospermia-like
11715280_s_at	8.03	0.000001	KRT17	keratin 17, type I
11756072_s_at	5.92	0.005264	SAA1	serum amyloid A1; serum amyloid A2; SAA2-SAA4 readthrough
11727092_x_at	5.53	0.000309	IL18	interleukin 18
11730408_a_at	5.48	0.00051	C19orf33	chromosome 19 open reading frame 33
11753131_x_at	4.81	0.015946	TM4SF1	transmembrane 4 L six family member 1
11717387_x_at	4.73	0.00164	MT1G	metallothionein 1G
11753130_at	4.52	0.008248	TM4SF1	transmembrane 4 L six family member 1
11755287_x_at	4.43	0.000674	KRT8	keratin 8, type II
11756334_x_at	4.37	0.00007	ANXA3	annexin A3
11753129_a_at	4.21	0.015138	TM4SF1	transmembrane 4 L six family member 1
11724283_a_at	4.08	0.000154	ANXA3	annexin A3
11718347_a_at	4.01	0.000348	S100P	S100 calcium binding protein P
11717385_a_at	3.84	0.00221	MT1G	metallothionein 1G
11719305_at	3.57	0.004942	SYNPO	synaptopodin
11727844_a_at	3.45	0.000574	KRT81	keratin 81, type II
11755325_s_at	3.36	0.00296	NPTX1	neuronal pentraxin I
11757396_s_at	3.08	0.000595	ANXA8	annexin A8; annexin A8-like 1; long intergenic non-protein coding RNA 842
11724159_x_at	3.08	0.000638	ECSCR	endothelial cell surface expressed chemotaxis and apoptosis regulator
11734418_s_at	2.96	0.000119	MAMDC2	MAM domain containing 2
11746917_s_at	2.86	0.000759	ANXA8	annexin A8; annexin A8-like 1; long intergenic non-protein coding RNA 842
11738054_a_at	2.8	0.000309	ITGB1BP2	integrin beta 1 binding protein (melusin) 2
11717576_a_at	2.78	0.000707	SFN	stratifin
11756316_a_at	2.75	0.016108	CHI3L1	chitinase 3-like 1 (cartilage glycoprotein-39)
11728596_a_at	2.71	0.003343	FAM180A	family with sequence similarity 180, member A
11733442_x_at	2.68	0.009126	SAA1	serum amyloid A1
11747398_a_at	2.64	0.000708	PLAT	plasminogen activator, tissue
11725695_s_at	2.62	0.000926	SDPR	serum deprivation response
11721357_at	2.62	0.001167	NMB	neuromedin B
11754461_s_at	2.61	0.000237	UCP2	uncoupling protein 2 (mitochondrial, proton carrier)
11747399_x_at	2.61	0.000443	PLAT	plasminogen activator, tissue
11755602_x_at	2.6	0.000409	ENO3	enolase 3 (beta, muscle)
11742082_a_at	2.58	0.004004	LCTL	lactase-like
11755208_a_at	2.57	0.000641	DNASE1L1	deoxyribonuclease I-like 1
11723019_at	2.57	0.006249	SUSD2	sushi domain containing 2
11719480_a_at	2.53	0.001942	CSTA	cystatin A (stefin A)
11715847_x_at	2.52	0.001012	PLAT	plasminogen activator, tissue
11749809_a_at	2.52	0.001477	PLAT	plasminogen activator, tissue
11755932_a_at	2.52	0.004499	HCLS1	hematopoietic cell-specific Lyn substrate 1
11717886_a_at	2.51	0.001044	PLAU	plasminogen activator, urokinase
11726809_x_at	2.45	0.00011	SLC12A8	solute carrier family 12, member 8
11721877_s_at	2.42	0.032646	MT1F	metallothionein 1F
11754545_x_at	2.4	0.000909	PLAT	plasminogen activator, tissue
11717154_a_at	2.39	0.001307	PLAU	plasminogen activator, urokinase
11724885_at	2.36	0.000714	CLIC3	chloride intracellular channel 3
11725694_at	2.34	0.008399	SDPR	serum deprivation response
11717017_a_at	2.29	0.003055	OCIAD2	OCIA domain containing 2
11724459_x_at	2.23	0.002011	MCAM	melanoma cell adhesion molecule
11731897_a_at	2.23	0.008893	PTHLH	parathyroid hormone-like hormone
11728397_at	2.23	0.047829	MT1M	metallothionein 1M
11762445_a_at	2.22	0.001184	LINC01588	long intergenic non-protein coding RNA 1588
11732321_a_at	2.2	0.000731	PLAT	plasminogen activator, tissue
11717041_at	2.2	0.023558	MTMR4	myotubularin related protein 4
11744067_s_at	2.18	0.001031	SYNGR2	synaptogyrin 2
11754184_a_at	2.18	0.001048	ALDH1A3	aldehyde dehydrogenase 1 family, member A3
11725855_s_at	2.17	0.004267	XAGE1B	X antigen family, member 1B; X antigen family, member 1E
11736061_a_at	2.16	0.001314	SERPINB5	serpin peptidase inhibitor, clade B (ovalbumin), member 5
11759423_at	2.14	0.003764	EPPK1	epiplakin 1
11737150_at	2.14	0.037566	IL26	interleukin 26
11755284_s_at	2.13	0.000763	TSPAN13	tetraspanin 13
11723805_a_at	2.12	0.000229	AKAP12	A kinase (PRKA) anchor protein 12
11756587_a_at	2.1	0.004	PTGDS	prostaglandin D2 synthase 21kDa (brain)
11740990_x_at	2.09	0.005501	HYAL1	hyaluronoglucosaminidase 1
11728398_x_at	2.09	0.029815	MT1M	metallothionein 1M
11725875_at	2.08	0.001219	WDR66	WD repeat domain 66
11722255_a_at	2.08	0.003273	RRAD	Ras-related associated with diabetes
11719121_a_at	2.08	0.012867	PPP1R14A	protein phosphatase 1, regulatory (inhibitor) subunit 14A
11728764_a_at	2.07	0.000526	PVRL4	poliovirus receptor-related 4
11755522_a_at	2.05	0.014466	LPXN	leupaxin
11733215_a_at	2.03	0.000011	STAT4	signal transducer and activator of transcription 4
11753736_s_at	2.03	0.024202	MT1JP	metallothionein 1J, pseudogene; metallothionein 1M; metallothionein 1 pseudogene 3
11755757_a_at	2.01	0.002924	SYNM	synemin, intermediate filament protein
11721304_at	2.01	0.027939	RAMP1	receptor (G protein-coupled) activity modifying protein 1
11730898_a_at	-2.01	0.000883	WAPL	WAPL cohesin release factor
11753968_a_at	-2.01	0.009979	SVEP1	sushi, von Willebrand factor type A, EGF and pentraxin domain containing 1
11753257_a_at	-2.01	0.014239	CXCL12	chemokine (C-X-C motif) ligand 12
11727672_a_at	-2.01	0.038642	MICAL2	microtubule associated monooxygenase, calponin and LIM domain containing 2
11737602_a_at	-2.02	0.014735	NLGN1	neuroligin 1
11748714_a_at	-2.03	0.008619	COL21A1	collagen, type XXI, alpha 1
11720819_s_at	-2.03	0.022427	CXCL12	chemokine (C-X-C motif) ligand 12
11720818_a_at	-2.07	0.008097	CXCL12	chemokine (C-X-C motif) ligand 12
11720508_at	-2.16	0.044294	FBN1	fibrillin 1
11715406_a_at	-2.19	0.020978	COL6A1	collagen, type VI, alpha 1
11755019_s_at	-2.21	0.022224	SCUBE3	signal peptide, CUB domain, EGF-like 3
11719970_a_at	-2.27	0.022329	PACSIN3	protein kinase C and casein kinase substrate in neurons 3
11732685_a_at	-2.38	0.008345	POGZ	pogo transposable element with ZNF domain
11718744_a_at	-2.68	0.0305	CXCL12	chemokine (C-X-C motif) ligand 12
11718889_x_at	-3.02	0.04201	PIP5K1A	phosphatidylinositol-4-phosphate 5-kinase, type I, alpha
AFFX-M27830_5_at	-4.5	0.045527		
